# Supplementary material for: Variation in outcome of invasive mechanical ventilation between different countries for patients with severe COVID-19: A systematic review and meta-analysis
Source: PLoS One. 2021 Jun 4;16(6):e0252760. doi: 10.1371/journal.pone.0252760 (PMC8177443; doi:10.1371/journal.pone.0252760)
Supplement: S1 Table — (DOCX) [file pone.0252760.s002.docx]

| Author | Newcastle Ottawa scale score; maximum 9 points (4,2,3) |
| --- | --- |
| [*Wang*](https://www.ncbi.nlm.nih.gov/pubmed/?term=Wang%20J%5BAuthor%5D&cauthor=true&cauthor_uid=32376592)*et al* (9) | **5 (2,1,2)** |
| [*Pedersen et*](https://www.ncbi.nlm.nih.gov/pubmed/?term=Pedersen%20HP%5BAuthor%5D&cauthor=true&cauthor_uid=32351196) *al (10)* | **5 (2,1,2)** |
| *Chen et al* (11) | *6 (2,1,3)* |
| *Yu et al (12)* | *4 (2,1,1)* |
| *Richardson et al (13)* | *7 (4,1,2)* |
| *Grasseli et al (14)* | *7 (4,1,2)* |
| *Zangrillo et al (15)* | *5 (2,1,2)* |
| *·     Bhatraju et al (16)* | *6 (2,1,3)* |
| *Kato et al (17)* | *5 (2,1,2)* |
| *Wu et al. (18)* | *6 (2,1,3)* |
| *Yang et al (19)* | *6 (2,1,3)* |
| *Ruan et al. (20)* | *6 (2,1,3)* |
| *Zhou et al (21)* | *6 (2,1,3)* |
| *Cummings et al (22)* | *7 (4,1,2)* |
| *Xu et al (23)* | *5 (2,1,2)* |
| *Argenziano et al (24)* | *7 (4,1,2)* |
| *Mitra et al (25)* | *5 (2,1,2)* |
| *Auld et a l(26)* | *7 (4,1,2)* |
| *Hur et al (27)* | *6 (3,1,2)* |
| *Petrilli et al (28)* | *7 (4,1,2)* |
| *Docherty et al (29)* | *7 (4,1,2)* |

**S1 Table : Quality of the included studies; NOS = Newcastle Ottawa scale**

**Maximum score 9: selection of study groups (four points); comparability of groups (two points); and ascertainment of exposure and outcomes (three points)**

*Stang A: Critical evaluation of the Newcastle-Ottawa scale for the assessment of the quality of nonrandomized studies in meta-analyses. Eur J Epidemiol 2010, 25: 603-605.*
