# Supplementary material for: Variation in outcome of invasive mechanical ventilation between different countries for patients with severe COVID-19: A systematic review and meta-analysis
Source: PLoS One. 2021 Jun 4;16(6):e0252760. doi: 10.1371/journal.pone.0252760 (PMC8177443; doi:10.1371/journal.pone.0252760)
Supplement: Attachment — Submitted filename: RRR Plos One.docx [file pone.0252760.s003.docx]

**Response to reviewers**

**Associate editor:**

Thank you for praising our manuscript and providing precise and constructive recommendations for revision

**Comment 1** Please attach a Supplemental file of the results a the quality assessment for each individual study assessed, reporting the outcome for each individual criteria considered.

Answer: A supplemental file is attached

**Comment 2:** We noticed you have some minor occurrence of overlapping text with the following previous publications, which needs to be addressed:

- https://associationofanaesthetists-publications.onlinelibrary.wiley.com/doi/full/10.1111/anae.15201

- https://www.atsjournals.org/doi/pdf/10.1164/rccm.202004-1385ED

- https://annalsofintensivecare.springeropen.com/articles/10.1186/s13613-020-00692-6

In your revision ensure you cite all your sources (including your own works), and quote or rephrase any duplicated text outside the methods section.

Changes: All mentioned sources have been addressed

**Reviewer 1**

Thank you for your valuable and constructive comments

**Comment 1:** I would suggest editing the introduction so to make it current: it should definitely be mentioned that vaccination has begun as a vaccine has been approved and more will follow.

Answer: The introduction has been edited and a paragraph about vaccination added.

**Comment 2:** A reference to an extremely recent meta-analysis titled Case Fatality Rates for Patients with COVID-19 Requiring Invasive Mechanical Ventilation. A Meta-analysis by Zheng Jie Lim et al. as well as discussion of its implications is warranted in the introduction section;

Answer 2: The reference has been added in the introduction and the implications of the study mentioned

**Comment 3:** It would be helpful to mention mortality rates after the initiation of mechanical ventilation for other conditions treated in the ICU other than the previous coronavirus diseases (eg. sepsis, pancreatitis, bacterial pneumonia, other viral pneumonias, COPD exacerbations) in the discussion. It is important to know whether the mortality of COVID19 when treated with mechanical ventilation is similar to other diseases which may be treated similarly in the ICU

Changes 3: A paragraph added in the discussion quoting mortality from other diseases treated in the ICU

**Comment 4:** I would also suggest doing a sensitivity analysis by including the 6 studies which included only patients with a definitive outcome. Another sensitivity analysis that could be attempted is the exclusion of studies with a small number (eg. less than 100) of patients with a definitive outcome. This may lead to a result with lower heterogeinty.

Changes 4: 2 new analysis have been added as required
